# Pianism: Performance Communication and the Playing Technique

**DOI:** 10.3389/fpsyg.2018.02125

**Published:** 2018-11-05

**Authors:** Barbara James

**Affiliations:** School of Human Movement and Nutrition Sciences, The University of Queensland, Brisbane, QLD, Australia

**Keywords:** piano technique, efficient playing movements, muscle mechanics, motor skills for expert performance, practice strategies

## Abstract

A pianist’s movements are fundamental to music-making by producing the musical sounds and the expressive movements of the trunk and arms which communicate the music’s structural and emotional information making it valuable for this review to examine upper-body movement in the performance process in combination with the factors important in skill acquisition. The underpinning playing technique must be efficient with economic muscle use by using body segments according to their design and movement potential with the arm segments mechanically linked to produce coordinated and fluent movement. Two physiologically and pianistically important actions proposed by early music scientists to deliver the keystroke involve dropping the hand from the shoulders toward the keys via a wave action with the joints activated sequentially, and forearm rotation to position the fingers for the keystroke, an action followed by the elbow/upper-arm rotating in the opposite direction. Both actions spare the forearm muscles by generating the energy needed in the larger shoulder muscles. The hand in the playing position has a curved palm through action of the metacarpal (knuckle) joints and curved fingers. Palm/finger posture controls sound quality from loud, high tempo sounds to a more mellow legato articulation, and to perform effectively the forearms should slope down toward the keyboard. The technique must be automatic through systematic practice which develops the motor skills for proficient playing, with practice duration tempered to reduce the risk of causing injury through overuse of the forearm muscles. Efficient movement patterns and strategic muscle relaxation which results in faster movement are realized only through extensive training. The constant movements of the head and trunk, and flowing arm movement with frequent hand lifts and rotational elbow movements, although generated in producing the playing technique, resonate with audience members who perceive them as expressive and thereby creating in them an empathic engagement with the music. It was proposed that music students be trained in the mechanical aspects of upper-body use in the playing technique, and practice strategies, with specialist pedagogy for children to develop motor skills for efficient playing, and training methods fostering an appreciation of the communicative aspects of music performance.

## Introduction

A pianist’s dynamic postures and movement patterns play a fundamental and functional role in the successful realization of a performance through their interaction in producing the playing movements generating the sounds, and the complementary expressive gestures conveying the composition’s structural architecture and emotional texture. Performance competence is built on the playing technique, with skilled players acquiring the motor skills to coordinate movement of a multi-joint limb to produce a fluent playing style. Early musical theorists realized the playing technique had not kept pace with the changes in the music environment which called for greater bodily participation (Matthay, 1903). Keyboards had evolved to have heavier action, increased resistance in the keys, wider keys to suit male concert pianists (Donison, 2000) resulting in an increased octave span by 25 mm, and larger concert spaces demanding louder playing. Because of these changes, it was hypothesized that the playing actions should not be focussed on hand/fingers but on the arm, introducing the concept of “*use of arm weight*” by dropping the arm from the shoulders (Matthay, 1903). This resulted in the realization that the technique needed to be efficient which implied making muscle use more economical by expending only the muscular energy needed for specific procedures, so that the energy load was manageable and the technique sustainable both during a performance and in the long term (Ortmann, 1929/1981). Deppe, in 1903 realized that as natural movements are curved, movement in a straight line, for example, negotiating the linear keyboard, additional muscular activity is needed to keep the hand on this plane, and the forearm rotating around its own axis would be more efficient in negotiating the keyboard (Gerig, 1974, p.334). This action was later considered the basis on which technique could be built, making it important pianistically and physiologically (Bernstein, 1967).

Music is a powerful communication mode, and during a performance a pianist conveys the music’s storyline to an audience through dynamic postures and body movements which generate the sounds and the expressive gestures which accompany them. As sound is fleeting, these movements conveying information about the pianist’s expressive intent to reinforce the impact of the sounds giving them significance and meaning (Munoz, 2007; Broughton and Stevens, 2009). The performer’s expressive playing increases audience appreciation of the performance influencing their judgment of the pianist’s skill and musicianship (Clarke, 2006) making it evident that the visual aspects of the postures and movements contribute to the way the performer and listeners perceive and mentally participate in a performance (Cole and Montero, 2007). With the advent of radio and recorded performance, music came to be viewed as a purely acoustic phenomenon (Thompson et al., 2005), however, recent research established that observers also depend on movement cues for their understanding of the musical ideas communicated in the unfolding sounds (Tsay, 2013). Pianists’ movements are important for musically-untrained observers who engage differently with the music from the musically-trained with the performer’s movement quality increasing the sensitivity of these observers to sound artifacts such as expressive temporal variation and timbre and helping to clarify perception of the emotion articulated (Vines et al., 2011).

The link between pianists’ playing movements and an expressive performance made it valuable for this interdisciplinary review to reflect on the function of body segments in providing a playing technique that allows the body to move in response to a score’s technical and expressive goals. It begins with sections on the: muscle mechanics and function of the upper-body segments in relation to the playing technique; the inter-segmental dynamics contributing to economic movement; implications for the sitting position in providing for the playing movements while allowing for spontaneous actions resulting from the musician’s ongoing interaction with the musical sounds. The review concludes with thoughts on training the next generation of professional pianists to reach a high standard of performance, while appreciating that the visual aspect of a pianist’s movement is important in performer-audience communication. The overall goal is to contribute to our understanding of the role of pianists’ movements in the process of preparing for and delivering a performance.

## Main Contribution

This section considers the playing technique, the movement potential and mechanics of the upper-body segments, the seated position allowing flexible function of the upper body, and the motor skills necessary for proficient delivery of the playing movements. The primary components of the musculoskeletal system are the: bones and joints which govern the direction of movement and shape of the trajectory, and; muscles, which differ in size and shape and endurance capacity, and provide the internal energy to generate movement which is moderated by *gravity*.

Despite the long-standing theoretical concepts involving the arm, it is only in this decade that performance neuroscientists have had the tools to analyze and quantify their benefits and offer a new understanding of how the upper-limb achieves the movement goals. The research referred to in this paper examined movement organization of the different factors affecting the playing technique.

### Muscle Mechanics and the Playing Technique

Muscular activity is the principal driver of the playing movement and an important variable is the inter-muscular difference in endurance capacity which is central to providing an efficient technique, and which varies directly with muscle cross-sectional area, and with the predominant fiber type (Herzog, 2000). The larger muscles of the trunk and shoulder girdle which move the shoulder/arm are comprised of “slow twitch” fibers which contain many blood capillaries making them able to keep using oxygen (from the atmosphere) for energy and they are more efficient for prolonged use when playing continuous keystrokes. The finer muscles of the forearm and hand consist of “fast twitch fibers” and contain relatively few blood capillaries so that when their energy supply is depleted, they are less able to access oxygen and with the resulting accumulation of waste products such as lactic acid and carbon dioxide causing discomfort, so they are best suited for short bursts of movement for strength or speed. Because playing actions are continuous and repetitive, it is better to generate energy for the keystroke from the shoulder muscles for transfer to the forearm/hand muscles. A problem with using the smaller forearm muscles is that they are prone to overuse with frequent and long-duration practice sessions when pianists are practicing for an audition or competition and performing rapid and forceful movements (Furuya et al., 2006).

The muscular energy needed for a procedure is moderated by *gravity*, the external force directed downward through the centre of the body where weight is evenly balanced. While playing, the balance changes constantly and gravitational force either increases or decreases the internally generated muscular force needed to make a movement or support a body part. Gravitational force can be utilized with vertical downward movements such as the downswing prior to the keystroke with a reduction in the finger-key force generated for the key-impact force (Furuya et al., 2009). Allied to gravity is the natural law stating that *for every action there is an equal but opposite reaction* (Newton’s third law of motion), and this becomes relevant when the fingers deliver a force to the keys, because after key-impact a rebound motion occurs initiating the lift for the next downswing, or if the fingers remain on the keys, the key force is absorbed by wrist undulation generated as a follow-through action.

### Use of Arm Weight

The synchronized system coordinating the motion of the multi-joint arm segments offered a viable framework referred to as the “arm-complex” by which to examine the playing technique referred to as “*use of arm weight*” to explain how the performer’s movements are organized. This unit extending from the sternum-clavicle junction (mid trunk) to the fingers is recognized biomechanically as a *kinetic chain* because of its action in producing a coordinated proximal-to-distal linked system in which the hand as the terminal segment can move freely thereby allowing versatile hand function including fine motor skill execution (Elliott et al., 1986). Through a “wave” action initiated by shoulder flexion, energy is transferred sequentially through the joints, with the energy generated in each joint additive, so that maximum momentum is achieved in the final finger segment for the force to be delivered in the finger/key impact (Glazier et al., 2003).

The downward motion of the relaxed arm enables muscular energy generated by the shoulder muscles to be delivered directly to the fingers to provide the force necessary for the finger/s to produce the required sound level and articulation (Furuya and Kinoshita, 2008). The shoulder motion results in forward movement of the upper-arm, with the forearm being thrust forward and the hand rotating forward, and with the fingers pointed downward to accomplish a more vigorous key depression (Kinoshita et al., 2007). This co-ordinated system, in combination with the downward force of gravity means that muscular energy generated by the shoulder muscles can be delivered directly to the keys, and with relaxed muscles, the forearm is in freefall increasing movement efficiency through the reduction of forearm muscle activity (Furuya et al., 2009).

### Forearm Rotation

The long bones of the forearm—*radius* (thumb side) and *ulna* (little finger side)—rotate about an imaginary axis to turn the hand inwards (pronation) or outward (supination) from the mid-line. Although the elbow and wrist move independently in lifting or lowering the forearm or hand, in rotation they are mechanically linked, making it impossible to rotate the forearm without also rotating the wrist/hand, and the relaxed elbow and upper-arm move laterally in the opposite direction, so that all the segments of the arm are linked. The rotational movement is important in positioning the hand/fingers at the right angle for finger/key impact thereby improving the efficiency of high-frequency repetitive keystrokes (Furuya et al., 2011) needed for the virtuoso piano performance so much in demand today (Hasanoglu, 2013).

### The Hand/Fingers and the Playing Movements

The hand must change its shape through the posture of the palm and fingers to strike piano keys in the most strategic way in producing the desired tempo and articulation. The curve across the palm formed through action of the metacarpal (MC) joints (knuckles) is very mobile and the palm can be either flat or curved with the fourth and fifth fingers descending to form a semicircle with the opposing thumb, while the second and third MC joints remain rigid (Kapandji, 1978, p.148). Longitudinally, the fingers curve downward from the MC joints, with the first joint of the four digits flexing or extending according to whether the fingers need to be flat or curved. In general, the ability of the palm and fingers to curve or flatten for key impact allows variability of movement patterns to produce a range of sound levels and articulation quality from *staccato* to *legato* (see Section “Communication and Sound Quality”). The curved hand helps the fingers reach different play positions, and the thumb being close to the keys means it can reach out to the next anchor point so the fingers can then move over to a new position. With the palm/fingers flattened the fingers can stretch to play chords or octaves, and on relaxing the stretch, the palm/fingers curve again automatically. When the hand and fingers are relaxed in a neutral position the fingers maintain a curved posture and with curved fingers on the keys, the fingers are functionally the same length (Kapandji, 1978, p.149).

### Relaxation of Muscles and Joints

Producing an efficient technique is reliant on having muscles without undue tension at strategic times, because with the arm free, movement can be produced faster if muscle contraction does not have to be overcome initially (Repp, 1993; Ross and Wakeling, 2016). With the fingers on the keys in the playing position, the elbow and wrist can relax, with finger/key impact made with the added weight of the arm passed smoothly from finger to finger during sequential key presses. Advantage can be taken of gravity when dropping the forearm/hand for finger/key contact, if the muscles relax during the downstroke so the forearm drops without the need of muscular action (Furuya et al., 2009), and similarly with trajectory landings, with these actions faster than those controlled by muscular action (Furuya et al., 2012). Directional changes in trunk movement such as surging and swaying, which is a fundamental element in expressive playing need also to have the trunk relaxed, with freedom of the neck and upper-back necessary also to facilitate greater ease for arm movements (Demos et al., 2014). The natural tonus of the muscles of the shoulder girdle and core muscles of the abdominal wall and vertebral column contribute to holding the trunk upright (Steinmetz et al., 2010). Strategic relaxation involved in an efficient technique is an unconscious process—a motor skill developed gradually through years of exposure to musical training.

### Implications for the Seated Position

**FIGURE 1:**
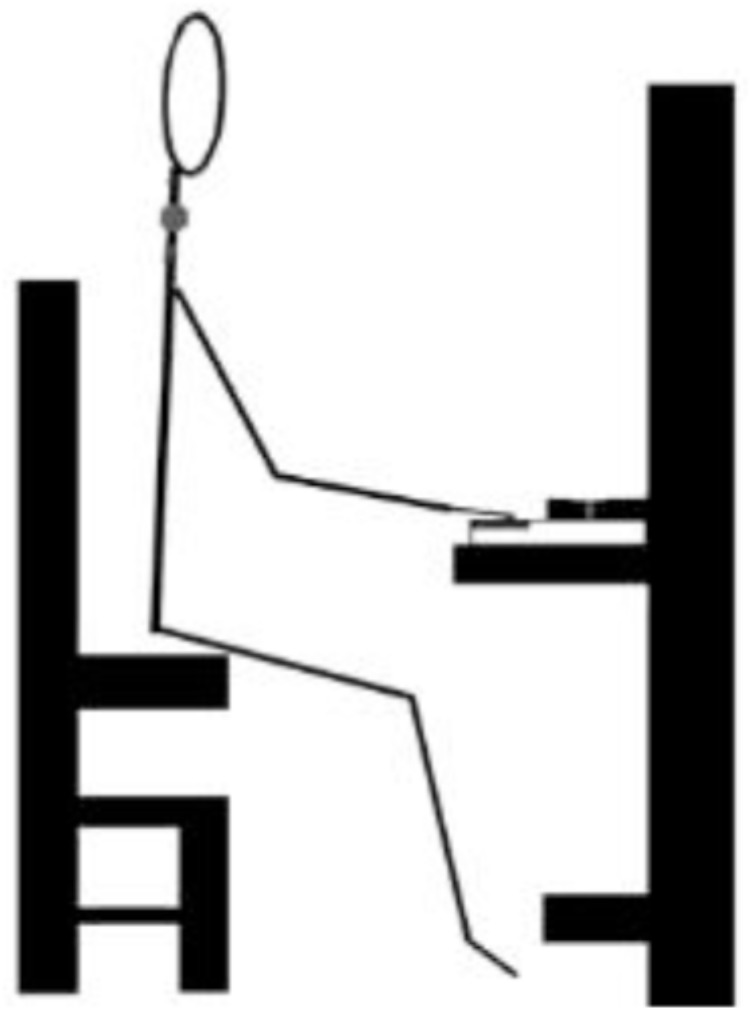
Reference sitting posture on front of piano stool, elbows extended about 110–120° so forearms slope down to the keyboard.

The whole body is involved in creating music by providing the playing actions, and a stable base for unrestricted upper-body movement. With the pelvis anchored on the piano bench, gravitational force transferred from the lower back and pelvis to the feet provides stability for dynamic trunk and arm movements, with the trunk swaying front to back, or side to side (Demos et al., 2014), and the arms having freedom to move in front of, across, and out to the sides of the body. With the trunk inclined toward the keyboard, the shoulder complex is well positioned to apply force to the fingers to maintain a stable position on the keyboard. The sitting height is determined by the position of the elbows which should allow the forearm to slope down to the keyboard with the angle between the upper-arm and forearm at about 100–110°. The reference sitting position is illustrated in Figure 1 below. With this orientation the biceps muscle is slightly stretched (Kapandji, 1978, p.93) affecting the elastic energy storage capacity of the muscle-tendon unit, with the muscle contracting faster through elastic recoil (Finni, 2001). Having the elbow higher than the wrist facilitates the transfer of energy down from the shoulders, and the hand/fingers are in the optimal position to perform effectively at all sound levels and tempi (Furuya et al., 2009). As well, the height of the trunk above the keyboard is important in maximizing the energy that can be delivered to the fingers with the forward swaying of the trunk and is very important for females with their reduced skeletal dimensions and muscle mass in comparison with males.

### Motor Skill, Practice and Automatic Playing Movements

Music performance is a complex sensorimotor behavior that becomes automated through involvement of all motor and sensory areas of the brain through systematic practice with auditory feedback. Pianists depend on highly developed motor skills for the acquisition of the novel motor and perceptual skills involved in encompassing a new score and in manipulating elements of music such as tempo, sound level, and tone timbre (Munoz, 2007) which are reliant on the organization of multi-joint movements in the hand and arm. Through constant practice, the playing movements are gradually consolidated into motor programs (muscle memories) to allow passages to be automatically, and when a movement sequence is repeated over time, a long-term muscle memory is created, eventually allowing it to be performed without conscious effort (Riemann and Lephart, 2002).

Motor sequences become automated through the repetition of passages, with the spatial patterns entrained so the hand “knows” how far to move, and the fingers are prepared for key contact, with pre-set joint angles and muscle force levels needed to produce the required tempo and sound (Rosenkranz and Rothwell, 2006). The automatic release of successful motor programs is necessary, so that during a performance, performers can trust their memories to work reliably under pressure (Chaffin and Imreh, 2002). The outcome of this process is that the actions shaping the score’s significant contours, tempo, dynamic and articulation or the expressive movements made in response to the sounds are embedded in the playing actions and integrated into the motor sequences producing the playing technique which means they can be reproduced (Jabusch et al., 2009). These dual roles are coordinated through unrestricted dynamic postures and movement patterns with the performer confident in letting the entrained motor sequences be produced automatically allowing the pianist to concentrate on the interpretation and expression of the musical ideas, while gaining confidence in their performance (Steinmetz, 2009). Motor programs expand to meet both technical and expressive goals and so are dynamic in responding to the player’s impulses and feelings (Schmidt and Lee, 2005).

During the early learning stage, practice at a slower tempo is more effective in facilitating movement control through constant feedback involving sensory information from *audition* and *touch*. When the tempo is later increased, movement patterns have greater accuracy through refinement of the movement patterns, and the playing movements are more efficient even though the maximum speed of the finger movements increases (Furuya et al., 2013). Playing movements made instinctively in response to the flow of the musical ideas allow a performer to relax and rely on the free flow of the movement (Parncutt and Troup, 2002) so attention can transfer from the movement specifics to the overall feel of the playing, with a changed focus of attention from “how” it is played to “what” is played (Chaffin and Imreh, 2002). Muscle memories associated with a certain score remain in the brain over time, and given the right cues, can be revived when practising a piece even after many years (Jabusch et al., 2009).

## Discussion

Musicianship involves a synthesis of many musical faculties to produce a coherent performance representing the performer’s musical ideas and fashioned by the movements which give life to a composition (Clarke, 2005). Music is closely linked to the body with the performer’s playing movements the mediator conveying the aesthetic information shaping the events in a score and giving the music its meaning. The pianist’s movement quality is the primary factor in creating effective musician-to-audience communication with the dynamic movement patterns providing the core of the music experience. The intimate connection between the performer and an artistic performance made it valuable for this theoretical review to reflect on the function of body segments in providing the playing technique and the complementary elements contributing to the delivery of an expressive performance. Preparing a score for performance involves an artistic process, so as well as the planned actions, the pianist needs a mental and physical readiness to respond to ideas/reactions arising while performing, so each presentation sounds fresh and spontaneous (Chaffin et al., 2004; Clarke, 2006).

### Communication and Sound Quality

Pianists both generate and react to the musical sounds which reflect their perception of the score’s structural framework and changing moods by varying the tempo, dynamics, and *ritardandi* to underscore the phrase structure and hierarchy and the expressive level (Vines et al., 2005). The quality of musicians’ movements supports communication by affecting the perception of musical properties, such as timbre, loudness, and pitch (Broughton and Stevens, 2009), and the amplitude and duration of musicians’ playing movements is helpful to music-naïve observers in their perception of the expressive intent communicated, and in discerning the duration of time-related aural events vital to the score interpretation such as accent notes and pauses (Schutz and Lipscomb, 2007). Tone timbre plays a key role in the communication of emotion and so it is vital to the expressiveness and overall affective quality of a music performance (Aho and Eerola, 2013).

Dexterous molding of the hand/fingers is important to producing the required sound quality because the way key force is transmitted affects audience-perception of sound level and quality (Furuya et al., 2012). A near vertical orientation of the final finger joints results in a rapid transfer of force to the key causing the perception of a louder tone through the instantaneous key movement and making it best suited to loud, fast playing. Conversely, with extended fingers, key contact is made with the fleshy finger pads allowing a larger surface area for force transmission resulting in a gradual transfer, lengthening the acoustic qualities of the sound, and making it preferable for the development of tone timbre to produce the mellow, full-bodied legato sound needed in many compositions (Furuya et al., 2012). Expressive variation in tone timber, an essential feature of the communication of emotion, results from the musician’s instinctive manipulation of touch through finger/key pressure intensity and duration allied with pedalling (Palmer, 1997; Bernays and Traube, 2011). The use of sound artefacts allows for spontaneity of musical expression and is related directly to the skill level and musicality of performers (Juchniewicz, 2012; Bernays and Traube, 2014) with variation in sound quality and expressive temporal variation avenues by which performers show their individuality through their creative response to the score’s notation (Repp, 1992; Thompson et al., 2005).

### Expressive Gestures: Interaction With the Playing Technique

Expressive body movements emerge spontaneously during music-making and act as a type of body language to extend the window of communication to reinforce and highlight the auditory events and clarify the expressive intentions of the music (Davidson, 1993; Rodger et al., 2012). Audience appreciation is influenced by head movements which provide key signals of expressive intent and the number of directional changes in trunk movement, such as swaying which is used almost universally by pianists to define cycles of tension indicating phrase boundaries and clarify the score’s structural architecture (Wanderley et al., 2005; Demos et al., 2014). Trunk/head movements increase in frequency as the emotional intent of the music becomes more intense (Davidson, 2012), and contribute to the playing technique through the transfer of momentum developed by the upper-body movement, and delivered to the fingers, increasing finger/key force needed for the keystroke in a large concert space requiring high sound-level playing (Furuya et al., 2010).

Arm and hand playing movements are viewed as transmitting grace and beauty, with audiences responding positively to the arm being active through frequent use of hand lifts, wrist undulation and elbow rotation described as “*elbow choreography*” (Bernstein, 1967) because it appears the elbow traces the contour of the music as it is played (Davidson, 2012). These arm/hand movements are generated in producing the playing technique with spontaneous hand lifts occurring as a reaction to the key force transmitted in the keystroke (Furuya et al., 2009). Elbow rotation resulting from hand/finger positioning for the keystroke is continuous with the serial changes in finger positioning (Furuya et al., 2011), and it is important that this movement is not inhibited by holding the elbows close to the trunk, a problem from early technique on lighter keyboards and which impedes elbow movement in response to the forearm rotation in reaching finger/key positions. Expressive gestures are performer-specific and are used across different genres, and there is a high degree of variability in the extent of the vocabulary of individual artists (Davidson, 2012).

### Practice and Playing-Related Injury

Practice is the principal activity of pianists: regular repetition of passages consolidates the muscle memories, and when the fingers know where they are going, space is created for pianists to concentrate on the sounds, evaluating their quality and fit with the dramatic concept of the score. The many hours of practice needed to meet a high playing standard leave the body vulnerable to playing-related injury (Zaza and Farewell, 1997), with the incidence and severity of injury related to the duration of practice sessions (Furuya et al., 2006). The primary risk factors include interactions between the playing technique, repertoire, and the fatigue related to extended activity in the muscles of the forearm/hand (Ramella et al., 2014) and which can result in inflammation of the nine flexor and six extensor tendons running through the carpal tunnel causing pressure on the median nerve and leading to the playing-related injury, “*carpal tunnel syndrome*” (Furuya et al., 2006).

Sustained high tempo playing can lead to pianists experiencing arm/hand pain (Furuya et al., 2006) with the decreased inter-note intervals hindering proper organization of the keystroke, resulting in increased use of the forearm muscles for the required finger/key force (Furuya et al., 2010). With keystroke frequency as much as 2,000 notes/min, the hourly rate is far higher than would be allowed in industry through occupational health and safety obligations applied to manual tasks such as continuous data entry (Russell, 2012). Also prevalent in pianists is discomfort/pain in the neck and upper back resulting from a flexed trunk/head posture distributing weight forward with compensatory activity in the muscles supporting the spine (Furuya et al., 2006). Consequently, the trapezius, a muscle group designed to hold the head up in alignment with the spine is stressed through stretched muscle fibres causing pain/discomfort, and with disruption of motor patterns degrading technique (Lourenço et al., 2011). Pianists practicing might maintain this posture for some time through focusing on the hands/fingers when practicing a new score, playing difficult technical passages, or performing rapid, repetitive movement requiring precision (Brandfonbrener, 1997). It can also indicate the playing technique is not automatic through deficiencies in the skill acquisition process due to giving up sight-reading the score too early, in favor of trying to memorize it, whereas the physical memory is as important as that in the central nervous system.

Continuous keystrokes make demands on concentration and it is important to curtail activity when concentration is diminished, or discomfort experienced (Furuya et al., 2012) because of the risk of a loss of touch precision (Fitts, 1954). For practice to be effective individual sessions should be distributed over time so coordinated efficient movement patterns are maintained (Barry and Hallam, 2002). Intervals between sessions can be used productively to relax the muscles and joints through stretching procedures involving shoulder and hip extension, to counteract their flexed habitual posture while playing. Also productive is mental rehearsal and score analysis, allowing the pianist the opportunity to concentrate on developing a musical image of the piece (Bravo and Fine, 2009). Choice of repertoire for a performance, sometimes involving three scores, must also be considered because it is essential that the type of playing movements needed is varied.

### The Pianist’s Playing Movements and Performance

Movement plays many roles affecting the preparation of a score for performance, with the visual aspect of the playing actions important to audience comprehension and enjoyment of the musical story, and when the musical sounds convey the same affective valence as the expressive movements, the emotional response is intensified by their concurrent presentation, an effect known as the *cross-modal bias* (Vines et al., 2011). When considering the function of movement, it became evident that the strategic use of inter-segmental dynamics allied with the relaxation of muscles not used in a procedure is important to an efficient playing technique, while at the same time appealing to the audience and increasing their pleasure in the performance. Observers respond positively to the flowing arm/hand movements resulting from the musician’s mastery of the technique of the arm falling from the shoulder and elbow rotation—a key variable in skilled performance—with audiences attracted by its continuous movement quality and highlighting the importance of the specialized motor skill for efficiency and aesthetic appeal (Furuya et al., 2011). These movements, which are central to a proficient playing technique, also resonate with audience members who perceive them in their “mind’s eye” as meaningful, with the affective messages they initiate establishing a rapport between performer and viewers, and drawing them into the music (Manley, 2013).

A musician’s sitting height is important, because it allows greater flexibility of upper-body movements to articulate the score’s expressive objectives and history informs us that Liszt through practical intuitive experience understood instinctively and recommended to his students that a higher seat be used so greater power in the arm could be generated to maximize the finger/key force, because if sitting too low the elbows do not have the same freedom (Gerig, 1974, p.185). Playing movements cannot be divided into being either sound producing, expressive, or “ancillary” as suggested by some authors (e.g., Wanderley et al., 2005) because as movement is fluid with one note blending into the next, it is difficult to tease the functions apart. An additional quality of the playing movements is that they can be affectively rewarding for the performer, and resonate with observers (Cole and Montero, 2007) highlighting that we have a cognitive ability to derive pleasure from something as abstract as movement in space (Christensen and Calvo-Merino, 2013).

### Implications for Performance Delivery

Movement is fundamental to music making with the audience informed of the musical themes by the acoustic and the visual cues derived from the pianist’s movements, so that when preparing for a performance, video recordings should be viewed to evaluate how the “big picture” is coming together with a seamless fusion of the fluent and expressive soundtrack with the flow of the dynamic movements, keeping in mind that musically-naïve participants make use of the visual cues for their perception of the music’s expressive intent. The pianist’s movements are also important in maintaining the attention and interest of the audience, making it doubly important for him/her to assess whether the movement quality of the expressive elements in the score matches that expressed in the sounds (Vines et al., 2011), and remembering that audience enjoyment is enhanced if the amplitude of the playing movements increases, because wide-ranging playing movements draw the interest of observers (Shoda and Adachi, 2012) and help with the perception of the duration of both sounds and silences which are important for audience comprehension and appreciation of the musical text (Margulis, 2007).

## Conclusion

Pianists shape musical works for performance through continuously changing body postures and upper-body movements with the structural elements and expressive features functioning as the musical goals to which the performer’s movements are directed, thereby engaging their body/brain in the act of music making. Unfortunately, the mechanics of the playing movements have not always received sufficient attention from music trainers, even though many problems in the pianist’s movement patterns affect the piano technique itself and contribute to playing-related injury involving the arm/hand, neck and upper-back. Ortmann (1929/1981) wondered why music academics were not curious about why the growing knowledge of the physiological aspects of human movement were being ignored, and today, almost a century later, he might find that there has in general been little change, with the result that many teachers are teaching as they were taught (Lister-Sink, 1994). Pianists suffer a high injury rate, the equivalent of that experienced in industry (Russell, 2012) and tertiary students deserve to be informed about the role of the upper body in producing the playing actions, so they can make better decisions about key aspects of their technique, practising strategies and the avoidance of injury. Another factor in injury prevention for pianists, may be the lack of a reporting mechanism within music teaching facilities for students to seek help, early because early symptoms may not interfere too much with playing ability, but they can exacerbate and follow a pianist through their careers, in some cases curtailing it.

It appears there is a mismatch in the relationship between pedagogy, technique, and playing-related injury, raising the question of the impact on students of how they are introduced in their early years to efficient playing procedures. Specialist pedagogy for children is necessary with training methods planned with consideration of the maturation of sensory faculties and body dimensions. Motor skills must be developed early to use a relaxed downswing which children learn easily (Furuya et al., 2009), and forearm rotation when first playing five-finger exercises. Sitting height is important for children and the piano should be adjusted to their proportions. Music students in tertiary training need movement analysis competence for their future careers as professional pianists or teachers with an informed view of the playing technique and with an understanding that pianists’ playing movements are vital in enabling effective communication of a score’s structural and expressive information. As music performance is multi-disciplinary, other people benefiting from piano technique- and injury-related knowledge include specialist researchers in related sub-disciplines and music generalists integrating science and technique to inform the music fraternity.

## Author Contributions

The author confirms being the sole contributor of this work and has approved it for publication.

## Conflict of Interest Statement

The author declares that the research was conducted in the absence of any commercial or financial relationships that could be construed as a potential conflict of interest.
